# Development of the Human Mycobiome over the First Month of Life and across Body Sites

**DOI:** 10.1128/mSystems.00140-17

**Published:** 2018-03-06

**Authors:** Tonya L. Ward, Maria Gloria Dominguez-Bello, Tim Heisel, Gabriel Al-Ghalith, Dan Knights, Cheryl A. Gale

**Affiliations:** a BioTechnology Institute, University of Minnesota, St. Paul, Minnesota, USA; b Departments of Biochemistry and Microbiology and Anthropology, Rutgers University, New Brunswick, New Jersey, USA; c Department of Pediatrics, University of Minnesota, Minneapolis, Minnesota, USA; d Bioinformatics and Computational Biology, University of Minnesota, Minneapolis, Minnesota, USA; e Department of Computer Science and Engineering, University of Minnesota, Minneapolis, Minnesota, USA; University of Colorado Denver

**Keywords:** ITS2, fungi, infant, microbiome, mycobiome

## Abstract

With the advent of next-generation sequencing and microbial community characterization, we are beginning to understand the key factors that shape early-life microbial colonization and associated health outcomes. Studies characterizing infant microbial colonization have focused mostly on bacteria in the microbiome and have largely neglected fungi (the mycobiome), despite their relevance to mucosal infections in healthy infants. In this pilot study, we characterized the skin, oral, and anal mycobiomes of infants over the first month of life (*n* = 17) and the anal and vaginal mycobiomes of mothers (*n* = 16) by internal transcribed spacer 2 (ITS2) amplicon sequencing. We found that infant mycobiomes differed by body site, with the infant mycobiomes at the anal sites being different from those at the skin and oral sites. The relative abundances of body site-specific taxa differed by birth mode, with significantly more *Candida albicans* fungi present on the skin of vaginally born infants on day 30 and significantly more *Candida orthopsilosis* fungi present in the oral cavity of caesarean section-born infants throughout the first month of life. We found the mycobiomes within individual infants to be variable over the first month of life, and vaginal birth did not result in infant mycobiomes that were more similar to the mother’s vaginal mycobiome. Therefore, although vertical transmission of specific fungal isolates from mother to infant has been reported, it is likely that other sources (environment, other caregivers) also contribute to early-life mycobiome establishment. Thus, future longitudinal studies of mycobiome and bacterial microbiome codevelopment, with dense sampling from birth to beyond the first month of life, are warranted.

**IMPORTANCE** Humans are colonized by diverse fungi (mycobiome), which have received much less study to date than colonizing bacteria. We know very little about the succession of fungal colonization in early life and whether it may relate to long-term health. To better understand fungal colonization and its sources, we studied the skin, oral, and anal mycobiomes of healthy term infants and the vaginal and anal mycobiomes of their mothers. Generally, infants were colonized by few fungal taxa, and fungal alpha diversity did not increase over the first month of life. There was no clear community maturation over the first month of life, regardless of body site. Key body-site-specific taxa, but not overall fungal community structures, were impacted by birth mode. Thus, additional studies to characterize mycobiome acquisition and succession throughout early life are needed to form a foundation for research into the relationship between mycobiome development and human disease.

## Abstract (precis)

Humans are colonized by diverse fungi (mycobiome), which have received much less study to date than colonizing bacteria. We know very little about the succession of fungal colonization in early life and whether it may relate to long-term health. To better understand fungal colonization and its sources, we studied the skin, oral, and anal mycobiomes of healthy term infants and the vaginal and anal mycobiomes of their mothers. Generally, infants were colonized by few fungal taxa, and fungal alpha diversity did not increase over the first month of life. There was no clear community maturation over the first month of life, regardless of body site. Key body-site-specific taxa, but not overall fungal community structures, were impacted by birth mode. Thus, additional studies to characterize mycobiome acquisition and succession throughout early life are needed to form a foundation for research into the relationship between mycobiome development and human disease.

## INTRODUCTION

Early-life microbial colonization plays an important role in the healthy development of an infant. Extensive epidemiological surveys of the human microbiome have improved our understanding of how factors such as birth mode, diet, and exposure to antibiotics shape the bacterial microbiome in early life (1). Recent studies have also begun to elucidate key members of the bacterial community that may play specific roles in the health outcomes of infants. For example, the genera *Lachnospira*, *Veillonella*, *Faecalibacterium*, and *Rothia* have been shown to be protective against asthma development if present in the fecal bacterial microbiome early in life (3 months of age) (2). In comparison to the infant bacterial microbiome, the structure and potential connections to health outcomes of the infant fungal microbiome (mycobiome) have received much less attention.

Recent characterization of the infant mycobiome through amplicon sequencing of the internal transcribed spacer (ITS) regions of the rRNA locus (ITS region 1 [ITS1] and/or ITS2) has shown infant gastrointestinal tracts to be colonized predominantly with *Saccharomyces*, *Candida*, and *Malassezia* genera (3–7). Similarly to colonizing bacteria, colonizing fungi have been linked to health outcomes, including early-life (1 to 11 months of age) fecal colonization, with *Candida* and *Rhodotorula* being linked to atopy and asthma (6). Additionally, targeted approaches have demonstrated direct transmission of specific fungal species from mother to infant (8, 9). However, how the very early stages of fungal colonization occur and whether there is birth mode-specific fungal community transmission from mother to child remain unknown. To gain a better understanding of early fungal community establishment, we characterized the skin, oral, and anal mycobiomes of a cohort of infants (*n* = 17) over the first 30 days of life through amplicon sequencing of ITS2. For comparison purposes, maternal vaginal and anal mycobiomes were also characterized. We measured differences in mycobiomes with respect to body site and maturational dynamics and for impact of birth mode on community composition.

## RESULTS

### Infant mycobiomes vary by body site.

**FIG 1:**
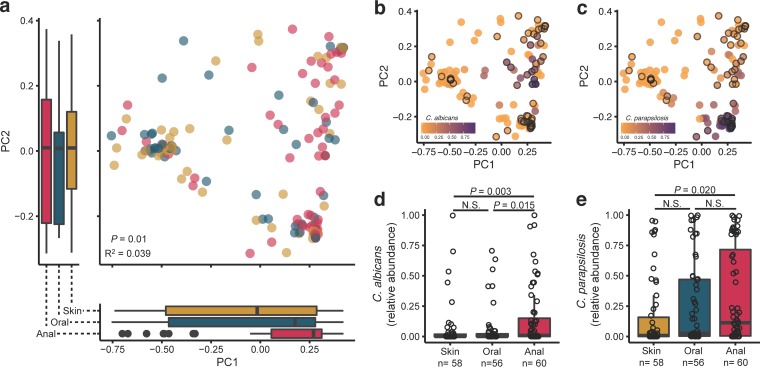
Infant mycobiomes vary by body site. (a) Principal-coordinate analysis of weighted UniFrac distances for infant skin, oral, and anal mycobiomes over the first 30 days of life. Box plots shown along each axis represent the median and interquartile range and indicate the distribution of samples along the given axis. Each point represents a single sample and is colored by body site as follows: skin, yellow (*n* = 58 samples); oral, teal (*n* = 56 samples); anal, pink (*n* = 60 samples). PERMANOVA values, *R*^2^ values, and *P* values are shown. (b and c) The same principal-coordinate analysis colored by the relative abundances of (b) *Candida albicans* and (c) *C. parapsilosis*, with anal samples denoted with a solid border. (d and e) Relative abundances of (d) *C. albicans* and (e) *C. parapsilosis* within the skin, oral, and anal mycobiomes of infants, as assessed by Wilcoxon rank sum tests with false-discovery-rate correction.

**FIG 2:**
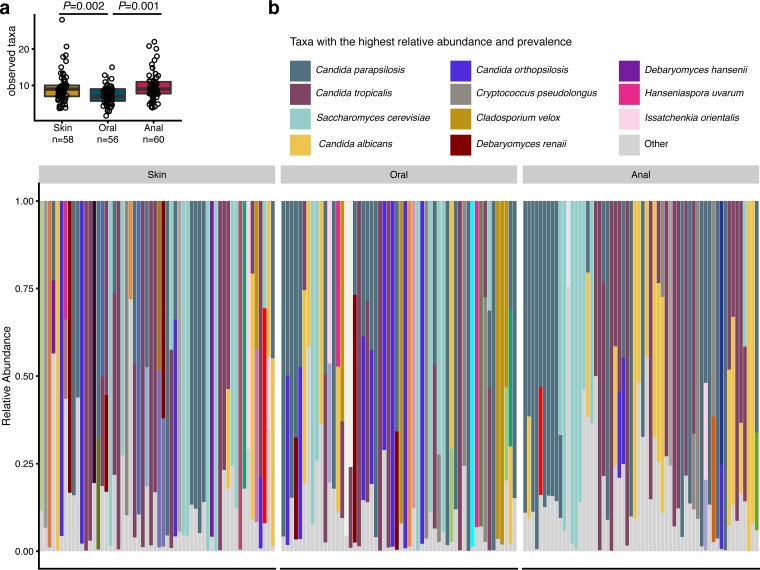
Specific taxa within the infant skin, oral, and anal mycobiomes. (a) Numbers of observed taxa within the skin, oral, and anal mycobiomes of infants, as assessed by Student’s *t* tests. (b) Relative abundances of fungal taxa within each body site. Each bar represents an individual sample, and samples are ordered by infant. The union of the 10 most abundant and 10 most common taxa is shown. “Other” represents taxa whose relative abundance is <10%. A full taxon legend is located in Fig. S1c.

To understand how early infant mycobiomes develop according to body site, we analyzed fungal DNA isolated from swabs of the skin, oral cavity, and anus (a mix of skin and feces) of infants at regular intervals over the first 30 days of life. Using principal-coordinate analysis of weighted UniFrac distances, infant anal mycobiomes clustered separately from oral and skin mycobiomes (Fig. 1a) (*P* = 0.01, *R*^2^ = 0.039). Clustering of anal samples was driven, in part, by the increased relative abundances of *Candida albicans* in anal mycobiomes versus skin mycobiomes (*P* = 0.003) and oral mycobiomes (*P* = 0.015), as well as increased abundances of *Candida parapsilosis* in anal samples versus skin samples (*P* = 0.020) (false-discovery rate [FDR] adjusted) (Fig. 1b to e). Oral mycobiome alpha diversity (observed species) was significantly lower than that of skin and anal mycobiomes using all samples across the first 30 days of life (*P* = 0.002 and 0.001, respectively) (Fig. 2a). However, comparing body sites at a specific time point (see Fig. S1a in the supplemental material), alpha diversity was not consistently lower in the oral mycobiome.

10.1128/mSystems.00140-17.1 FIG S1  Alpha diversity and taxa of maternal and infant mycobiomes over the first 30 days of life. (a) Total numbers of observed species in each body site per day (for the infants, total *n* = 58 skin samples, total *n* = 56 oral samples, and total *n* = 60 anal samples; for the mothers, *n* = 16 vaginal and 15 anal samples) were compared within each sampling day by the use of pairwise Wilcox tests and false-discovery-rate correction. No tests yielded a significant difference. (b) Relative abundances of fungal taxa within each maternal body site. Each bar represents an individual sample, and samples are ordered by mother. (c) Full taxon legend for Fig. 2b. “Other” represents taxa whose relative abundance is <10%. Download FIG S1, PDF file, 0.3 MB. Copyright © 2018 Ward et al. 2018 Ward et al. This content is distributed under the terms of the Creative Commons Attribution 4.0 International license.

Overall, infant mycobiomes tended to be dominated by relatively few taxa (Fig. 2b). The taxa of highest prevalence and relative abundance across all infant body sites sampled were *C. parapsilosis*, *C. tropicalis*, *Saccharomyces cerevisiae*, *C. albicans*, *C. orthopsilosis*, *Cryptococcus pseudolongus*, *Cladosporium velox*, *Debaryomyces renaii*, *D. hansenii*, *Hanseniaspora uvarum*, and *Issatchenkia orientalis* (also known as *C. krusei* [10, 11]) (Fig. 2b; see also Tables S1 and S2 in the supplemental material). Among the body sites, the most abundant and/or prevalent taxa were *C. tropicalis*, *C. parapsilosis*, *S. cerevisiae*, *C. albicans*, and *C. orthopsilosis* in the infant skin mycobiome; *C. parapsilosis*, *C. tropicalis*, *S. cerevisiae*, *C. orthopsilosis*, *C. albicans*, and *Cladosporium velox* in the infant oral mycobiome; and *C. parapsilosis*, *C. tropicalis*, *C. albicans*, *S. cerevisiae*, *C. orthopsilosis*, and *Cryptococcus pseudolongus* in the infant anal mycobiome (Tables S1 and S2). Similarly, the maternal mycobiomes (vaginal and anal) were also often dominated by a single taxon (Fig. S1b), with *C. albicans* being the most abundant (>20% in relative abundance) in the vaginal mycobiome and *C. albicans*, *S. cerevisiae*, and *C. parapsilosis* being the most abundant in the maternal anal mycobiome (Table S1). Of note, all of the major fungal taxa presented here have been previously reported to be human-associated taxa, and many are opportunistic pathogens in the setting of a compromised immune system (12).

10.1128/mSystems.00140-17.6 TABLE S1  Top five most abundant infant and maternal fungal taxa by body site. Download TABLE S1, DOCX file, 0.1 MB. Copyright © 2018 Ward et al. 2018 Ward et al. This content is distributed under the terms of the Creative Commons Attribution 4.0 International license.

10.1128/mSystems.00140-17.7 TABLE S2  Top five most prevalent infant and maternal fungal taxa by body site. Download TABLE S2, DOCX file, 0.1 MB. Copyright © 2018 Ward et al. 2018 Ward et al. This content is distributed under the terms of the Creative Commons Attribution 4.0 International license.

### Infant mycobiomes are individualized and variable.

**FIG 3:**
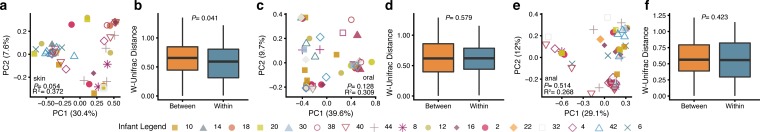
Infant mycobiome dynamics during the first 30 days of life. Data represent results of principal-coordinate analysis (a, c, and e) of weighted UniFrac (W-Unifrac) distances and median within-infant and between-infant weighted UniFrac distances (b, d, and f) for (a and b) skin, (c and d) oral, and (e and f) anal mycobiomes over time. Principal-coordinate analysis data (individual infant data noted by distinct colored shape; see legend) were tested with PERMANOVA; *R*^2^ values and *P* values are shown. Distances were compared using a Wilcoxon rank sum test (*n* = 17 infants).

Skin mycobiomes showed within-infant similarity over time, demonstrated by lower within-infant weighted UniFrac distances than between-infant distances (*P* = 0.041), as well as significant clustering of weighted UniFrac distances according to infant (permutational multivariate analysis of variance [PERMANOVA]; *P* = 0.054, *R*^2^ = 0.372) (Fig. 3a and b). The oral and anal mycobiomes of infants, however, exhibited high intraindividual variability for beta diversities over time with a lack of clustering by infant over time and similar average within-infant and between-infant weighted UniFrac distances (*P* > 0.05) (Fig. 3c to f). No individual infant’s mycobiome showed a clear trajectory toward a mature or distinct state during the first 30 days of life, regardless of body site, as demonstrated by the lack of a decrease in UniFrac distances from the same infant's day 30 sample over time (*P* > 0.05) (Fig. S2a to c).

10.1128/mSystems.00140-17.2 FIG S2  Infant mycobiome maturation over the first 30 days of life. Data represent the distance (weighted UniFrac) of each infant mycobiome from the corresponding day 30 mycobiome, according to body site. (a) Skin mycobiomes. (b) Oral mycobiomes. (c) Anal mycobiomes. *n* = 11 infants. *P* value data represent group-wise averages of permuted Spearman correlation test statistics controlled by subject. The dotted lines represent a summary linear model that controls for subject, with *R*^2^ reported. Download FIG S2, TIF file, 1 MB. Copyright © 2018 Ward et al. 2018 Ward et al. This content is distributed under the terms of the Creative Commons Attribution 4.0 International license.

**FIG 4:**
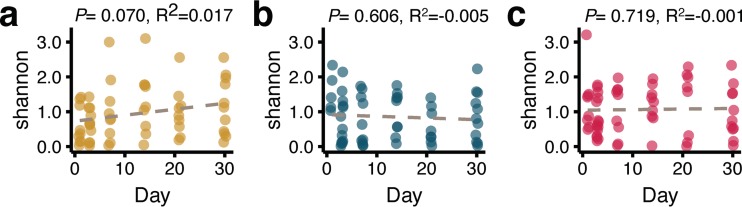
Infant alpha diversity over time. Shannon index values corresponding to fungal OTUs from (a) skin, (b) oral, and (c) anal mycobiomes are presented (*n* = 17 infants). *P* values represent group-wise averages of permuted Spearman correlation test statistics controlled by subject. The dotted lines represent a summary linear model that controls for subject, with *R*^2^ reported.

Infant and adult (maternal) mycobiomes were similar to each other with respect to alpha and beta diversity measures. The total numbers of observed taxa were similar for infant and adult samples for all infant sample collection time points, regardless of body site (Fig. S1a). Over the first 30 days of life, alpha diversities (Shannon index values) of infant mycobiomes did not significantly change (Fig. 4). For skin mycobiomes, there was a trend toward increasing diversity over time, although the data did not reach statistical significance (*P* = 0.070) (Fig. 4a). Infant anal mycobiome beta diversity was similar to that of adults (*P* = 0.369, *R*^2^ = 0.014) (Fig. S3a), with infant mycobiomes maintaining a similar distance from adult mycobiomes over the first 30 days of life (*P* = 0.121) (Fig. S3b).

10.1128/mSystems.00140-17.3 FIG S3  Diversity in infant and maternal anal mycobiomes. (a) Principal-coordinate analysis of weighted UniFrac distances of maternal (gray) and infant (pink) anal mycobiomes over the first 30 days of life. PERMANOVA values, *R*^2^ values, and *P* values are shown. (b) Distance (weighted UniFrac) of each infant anal mycobiome from the corresponding adult anal mycobiome, over the first 30 days of life (*n* = 17 infants and 16 mothers). The dotted lines represent a summary linear model that controls for subject, with *R*^2^ reported. The *P* value data represent group-wise averages of permuted Spearman correlation test statistics controlled by subject. Download FIG S3, TIF file, 0.9 MB. Copyright © 2018 Ward et al. 2018 Ward et al. This content is distributed under the terms of the Creative Commons Attribution 4.0 International license.

### Birth mode affects the relative abundances of specific taxa but not overall fungal community structures.

**FIG 5:**
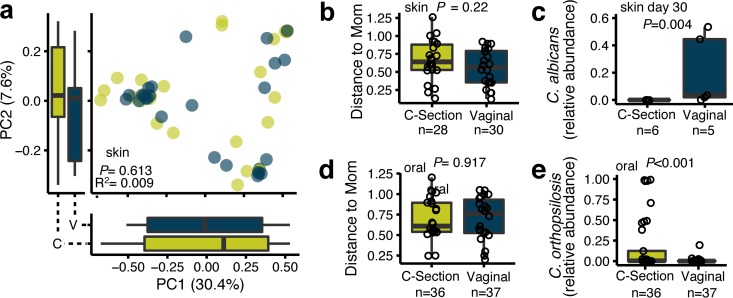
Infant skin and oral mycobiomes by birth mode. (a) Principal-coordinate analysis of weighted UniFrac distances of infant skin mycobiomes colored by birth mode (vaginal = blue, *n* = 30 samples; caesarean section = green, *n* = 28 samples). PERMANOVA values, *R*^2^ values, and *P* values are shown. (b and d) Weighted UniFrac distances of infant (b) skin and (d) oral mycobiomes from the mother’s vaginal mycobiome by birth mode (paired analysis), as assessed by a Wilcoxon rank sum test (*n* = 7 vaginal birth families and 9 caesarean section birth families across all time points). (c and e) Relative abundances of (c) *Candida albicans* in infant skin on day 30 according to birth mode and (e) *C. orthopsilosis* in the oral cavity across all time points, as assessed with a Wilcoxon rank sum test (false discovery rate corrected; *n* = 5 vaginally born infants, *n* = 6 caesarean section-born infants).

To examine whether early-life mycobiomes are impacted by birth mode, we first analyzed skin fungal communities for differences in alpha and beta diversity and for differentiated taxa in infants born vaginally compared to those born by caesarean section. Note that the effect of caesarean section represents a composite of the effects of lack of labor, perinatal antibiotics, and bypassing of the vaginal canal (Table 1). As shown by weighted UniFrac distances and principal-coordinate analysis, infant skin mycobiomes did not cluster according to birth mode (*P* = 0.613, *R*^2^ = 0.009) (Fig. 5a), and an infant’s skin mycobiome was not more similar to the mother’s vaginal or anal mycobiome than to a randomly chosen mother’s mycobiome (Fig. S4a). Similar findings were also obtained using Bray Curtis and unweighted UniFrac distances (data not shown).

10.1128/mSystems.00140-17.4 FIG S4  Infant mycobiome similarity to maternal mycobiome. Data represent the distance (weighted UniFrac) of each infant’s sample from the mother’s anal or vaginal sample (paired; orange) or from a permuted randomly chosen mother's sample (random; blue) for the infant’s skin (a), oral (b), and anal (c) mycobiomes, tested with Wilcoxon rank sum tests (*n* = 16). Download FIG S4, TIF file, 1.1 MB. Copyright © 2018 Ward et al. 2018 Ward et al. This content is distributed under the terms of the Creative Commons Attribution 4.0 International license.

**TABLE 1:** Participant data^a^

Family ID	Postnatal collection days			Mother sample collection sites	Perinatal antibiotics	Maternal group B *Streptococcus* status	Birth mode	Infant diet
	Skin samples	Anal samples	Oral samples					
2	3, 7, 14, 21, 30	3, 7, 14, 21, 30	3, 7, 14, 21, 30	Vaginal, anal	Cephalosporin	Negative	C-section	Mixed breast and formula
3	3, 7, 14, 21, 30	3, 7, 14, 21, 30	3, 7, 14, 21, 30	Vaginal, anal	Cephalosporin	Negative	C-section	Mixed breast and formula
4	1, 3	1, 3	1, 3	Vaginal, anal	Cephalosporin	Negative	C-section	Mixed breast and formula
5	3, 7, 14, 21, 30	3, 7, 14, 21, 30	3, 7, 14, 21, 30	Vaginal, anal	Cephalosporin	Negative	C-section	Exclusive formula
6	7, 14, 21, 30	3, 7, 14, 30	7, 14, 21, 30	None	Cephalosporin	Negative	C-section	Exclusive breastfeeding
7	3	3	3	Vaginal, anal	Cephalosporin	Negative	C-section	Exclusive formula
8	3, 7, 14, 21, 30	3, 7, 14, 21, 30	3, 7, 14, 21, 30	Vaginal, anal	Cephalosporin	Negative	C-section	Mixed breast and formula
9	3	3	3	Vaginal, anal	Cephalosporin	Negative	C-section	Mixed breast and formula
10	3, 7, 14, 21, 30	3, 7, 14, 21, 30	3, 7, 14, 21, 30	Vaginal, anal	Cephalosporin	Positive	C-section	Mixed breast and formula
11	1, 3	1, 3	1, 3	Vaginal, anal	Cephalosporin	Positive	C-section	Mixed breast and formula
15	1, 3	1, 3	1, 3	Vaginal, anal	Penicillin	Positive	Vaginal	Mixed breast and formula
16	1, 3, 7, 14, 21, 30	1, 3, 7, 14, 21, 30	1, 3, 7, 14, 21, 30	Vaginal, anal	None	Negative	Vaginal	Mixed breast and formula
19	1, 3	1, 3	1, 3	Vaginal, anal	None	Negative	Vaginal	Mixed breast and formula
20	1, 3, 7, 14, 21, 30	1, 3, 7, 14, 21, 30	1, 3, 7, 14, 21, 30	Vaginal, anal	None	Negative	Vaginal	Exclusive breastfeeding
21	1, 3, 7, 14, 30	1, 3, 7, 14, 30	1, 3, 7, 14, 30	Vaginal, anal	None	Negative	Vaginal	Exclusive breastfeeding
22	1, 7, 21, 30	1, 7, 21, 30	1, 7, 21, 30	Vaginal, anal	None	Negative	Vaginal	
1	1, 7, 14, 21, 30	1, 3, 7, 14, 21, 30	1, 3, 7, 14, 21, 30	Vaginal, anal	None	Negative	Vaginal	Exclusive breastfeeding

a ID, identifier; C-section, caesarean section.

To test whether this lack of differentiation by birth mode was due to high vaginal mycobiome variability across mothers, we tested for differences in the average distance of an infant’s skin sample data from the mother’s vaginal sample data. Infants born vaginally did not have a skin mycobiome that was more similar to the mother’s vaginal mycobiome than those born by caesarean section, although a nonsignificant trend was observed (*P* = 0.22) (Fig. 5b). This held true regardless of the infant sampling day or the beta diversity metric used. Skin mycobiome alpha diversities were also not significantly different with respect to birth mode for any time point within the first 30 days of life but did trend toward increasing over time in vaginally born infants but not in caesarean section-born infants (*P* = 0.071, and *P* = 0.331, respectively) (Fig. S5a). Interestingly, *C. albicans*, a common fungus inhabiting the vagina (13), had significantly higher relative abundance in skin mycobiomes of 30-day-old infants born vaginally than in skin mycobiomes of those born by caesarean section (FDR-adjusted *P* = 0.004) (Fig. 5c).

10.1128/mSystems.00140-17.5 FIG S5  Infant mycobiome characteristics according to birth mode. (a) Data corresponding to the Shannon index values determined for the skin, oral, and anal mycobiomes for caesarean section (green) and vaginally born (blue) infants over time are shown (*n* = 17 infants). The dotted lines represent a summary linear model that controls for subject, with *R*^2^ reported. *P* value data represent group-wise averages of permuted Spearman correlation test statistics controlled by subject. *t* tests were also performed on each sampling day to test for differences across birth modes. The only significant difference within day, after false-discovery-rate correction, is shown for the anal samples on day 21 (dotted box). (b) Principal-coordinate analysis of oral weighted UniFrac distances of vaginally born (blue, *n* = 26) and caesarean-section-born (green, *n* = 30) infants over the first 30 days of life. (c) Principal-coordinate analysis of anal weighted UniFrac distances of vaginally born (blue, *n* = 28) and caesarean-section-born (green, *n* = 32) infants over the first 30 days of life. (d) Weighted UniFrac distance of infant anal mycobiomes from the mother’s vaginal mycobiome by birth mode (paired analysis), as assessed by a Wilcox test (*n* = 7 vaginal birth families and 9 caesarean section birth families, with samples from all time points). PERMANOVA values, *R*^2^ values, and *P* values are shown for principal-coordinate analysis. Download FIG S5, PDF file, 0.5 MB. Copyright © 2018 Ward et al. 2018 Ward et al. This content is distributed under the terms of the Creative Commons Attribution 4.0 International license.

For oral mycobiomes, birth mode did not significantly impact the alpha diversity trajectory over time (caesarean section *P* = 0.238; vaginal *P* = 0.873) (Fig. S5a) or beta diversity clustering (*P* = 0.261, *R*^2^ = 0.022) (Fig. S5b). The oral mycobiome of an infant was also not more similar to that of the mother than to that of a randomly chosen mother (Fig. S4b) or to the mother’s vaginal mycobiome among the infants born vaginally than among those born by caesarean section (*P* = 0.917) (Fig. 5d). Caesarean section-born infants, however, did have a significantly higher relative abundance of *Candida orthopsilosis* than infants born vaginally (FDR-adjusted *P* = 0.001) (Fig. 5e).

For anal mycobiomes, alpha diversity was significantly higher among the infants born by caesarean section than among those born vaginally, but only for day 21 of life (*P* < 0.001), and did not significantly increase or decrease over time regardless of birth mode (*P* = 0.099 for birth by caesarean section; *P* = 0.973 for vaginal birth) (Fig. S5a). Birth mode did not significantly affect the beta diversity of the infant anal mycobiome (*P* = 0.97, *R*^2^ = 0.002) (Fig. S5c), and the anal mycobiome of an infant was not more similar to the mother’s mycobiome than to that of a randomly chosen mother (Fig. S4c) or to the mother’s vaginal mycobiome among the infants born vaginally than among those born by caesarean section (*P* = 0.513) (Fig. S5d).

## DISCUSSION

### Infant mycobiomes and body site differentiation.

Overall, the early infant mycobiomes consisted of a few (generally <16 per sample) unique operational taxonomic units (OTUs) and taxa, with individual mycobiomes often being dominated by one specific taxon (Fig. 2b). The finding of low fungal richness reported here is supported by a recent study of the infant fecal mycobiome which detected low fungal biomass and, on average, fewer than 12 fungal OTUs per sample over the first 2 years of life (7, 14). The most prevalent and abundant taxa across all infant body sites were primarily *Candida* species, with *C. parapsilosis* and *C. tropicalis* having the highest combined relative abundances (37%, 39%, and 56% of the skin, oral, and anal fungi, respectively; see Table S1 in the supplemental material). *C. albicans* and *S. cerevisiae* were also prominent (up to 15% relative abundance), depending on the body site. The detection of these species was expected, as they are well-known human-associated fungi. Previous analyses of skin-associated fungi showed adults to be predominantly colonized with the genus *Malassezia*, as well as being colonized with other genera, such as *Candida* and *Saccharomyces* (15, 16). Here, *Malassezia* was also detected in the infant skin mycobiome but accounted for only 2% of the relative abundance. This result is supported by the finding of lower relative abundance of *Malassezia* in the skin of children than in that of adults as shown by next-generation sequencing (17), PCR-based (18, 19), and culture-based (20, 21) approaches. The oral mycobiome of adults has previously been reported to be predominantly composed of species of the *Candida* (22) and *Malassezia* (23) genera. Previous exploration of the oral mycobiome of infants, however, has been restricted to the use of *Candida*-specific culturing, demonstrating the presence of numerous *Candida* species such as *C. albicans*, *C. parapsilosis*, *C. krusei*, *C. guilliermondii*, *C. geocandidum*, and *C. tropicalis* (24, 25). Similarly, we found the *Candida* species *C. parapsilosis*, *C. tropicalis*, and *C. orthopsilosis*, as well as *S. cerevisiae*, to be the most relatively abundant and prevalent fungal species in infant oral mycobiomes during the first month of life (Tables S1 and S2). Among the anal samples, *C. parapsilosis*, *C. tropicalis*, *C. albicans*, and *S. cerevisiae* were common and had the highest relative abundances (Table S1), consistent with previous studies of the infant fecal mycobiome, which reported *Saccharomyces* and *Candida* to be the predominant genera as well as the presence of other minor members (3, 5–7). Because the data presented here are based on relative abundance, which could potentially be impacted by ITS2 copy number, future studies highlighting the relative and total abundances of fungal taxa in infants are warranted.

The issue of whether or not immediate (at birth) and early-life body site differentiation of the bacterial microbiome exists is controversial. Some reports state that there is a detectable difference immediately after birth, when the infant meconium is significantly different in bacterial composition from skin and oral sites (26). Other reports, however, state that infant skin, oral, and rectal bacterial microbiomes cluster primarily according to delivery mode (27). Differentiation of the bacterial microbiome continues over time for skin, oral, and anal sites, with some studies showing detectable differences among all three sites as early as 4 to 6 weeks after birth (26, 28). Here, early-life anal mycobiomes of infants were found to be different from oral and skin mycobiomes and this was driven, in part, by the significantly higher relative abundances of *C. albicans* and *C. parapsilosis* in anal samples than in oral and skin samples (Fig. 1). Higher relative abundances of these species in the anal mycobiome may have been due, in part, to transition of the gut from an aerobic to an anaerobic state during the first weeks of life, favoring the initial expansion of opportunistic *Candida* species (7). For example, *C. albicans* is the most common fungal colonizer and pathogen of infants, likely due to its abilities to rapidly adapt to environmental changes through yeast-to-hypha morphogenesis, to adhere to human epithelial and endothelial cells, and to form biofilms (29, 30). Additionally, expansion of abundances of *C. albicans* and *C. parapsilosis* in the skin and oral mycobiomes may be limited by the presence of specific resident bacteria in these areas, as demonstrated by the ability of abundant oral taxa *Streptococcus* and *Lactobacillus* (31) to prevent the growth of (32) and to out-compete (33–35) *C. albicans*. Due to the small sample size and lack of bacterial sequencing in this pilot study, the mechanism determining fungal mycobiome differentiation by body site should be further explored with larger studies that include both bacterial and fungal sequencing.

### Birth mode comparisons.

The bacterial microbiome is highly variable with respect to birth mode; beta diversity differences are detectable immediately following birth (for skin and oral sites), and the initially higher fecal alpha diversity seen with caesarean section-born infants decreases over the first month of life to below that seen with vaginally born infants (26, 36). Many studies have also reported higher levels of specific taxa, such as *Bacteroides*, in the fecal bacterial microbiome of vaginally born infants than in the bacterial microbiome of those born by caesarean section (27, 36, 37). For the mycobiome, we did not detect differences in beta diversity with respect to birth mode over the first month of life regardless of sampling site (Fig. 5; see also Fig. S5 in the supplemental material). Mycobiome alpha diversities also did not significantly increase or decrease over the first month of life, regardless of birth mode and body site (Fig. S5a), which may have been due to the use of a relatively consistent food source of either breast milk or formula. Interestingly, the skin mycobiome of vaginally born infants did contain a higher relative abundance of *C. albicans* than that of caesarean section-born infants at 30 days of life (Fig. 5c), suggesting that either birth mode or perinatal antibiotics affect colonization by specific fungal species. A delayed increase in vagina-associated taxa in vaginally born infants was also previously seen for bacteria, albeit at much later times, where the number of maternal vagina- and infant stool-shared operational taxonomic units (OTUs) for vaginally born infants peaked between 18 and 24 months of life (36). Additionally, vertical transmission of specific *C. albicans* strains from mother to infant has been shown previously using culture-based and sequence-based (restriction fragment length polymorphisms) approaches (8). Due to the small sample size used here, future longitudinal studies of infants with larger cohorts that extend beyond 30 days after birth are needed to definitively determine if abundances of vagina-associated fungal taxa, including *C. albicans*, increase in the mycobiomes of infants born vaginally. Of note, a recent report showed low rates of *C. albicans* transmission from mother to infant, suggesting environmental as well as maternal sources for infant mycobiome establishment (38). Our results support the need for analyses for other mycobiome sources, as the average distance of an infant’s mycobiome from that of its mother was not significantly lower for infants born vaginally than for those born by caesarean section (Fig. 5b and d; see also Fig. S5d). Because maternal samples in this study were taken immediately following birth (after passage of amniotic fluid, the baby, and the placenta in cases of vaginal delivery), similarities between mother and infant mycobiomes could have been impacted by these factors. If mothers had been sampled just prior to delivery, perhaps the similarities between the mother and infant mycobiomes would have been higher.

Vertical transmission of skin-associated fungal species of the *Malassezia* genus from mothers to their caesarean section-born infants as determined using a targeted PCR-based approach was previously shown (9). We did not detect a significant increase in the relative abundances of skin-associated *Malassezia* species in the skin mycobiomes of infants born by caesarean section compared to vaginally born infants (data not shown). Although the maternal skin mycobiome was not analyzed here, based on the extensive evidence indicating the presence of *Malassezia* in the adult skin mycobiome (15, 16, 39, 40), the lack of increased skin-associated *Malassezia* abundance in caesarean section-born infants is not likely due to low maternal *Malassezia* levels. It is possible that *Malassezia* remains in low relative abundance within the first month of life, suggesting that, similarly to *C. albicans* colonization, *Malassezia* colonization may represent a mixture of maternal and environmental colonization sources. Additionally, because *Malassezia* species are basidiomycetous fungi, their low relative abundance in our results may have been due to our use of the ITS2 region, which may be biased toward the amplification of ascomycetous fungi (41, 42), for taxonomic classification.

The oral mycobiome of infants born by caesarean section contained a higher relative abundance of *C. orthopsilosis* than that of vaginally born infants over the first month of life (Fig. 5e). *C. orthopsilosis* was previously characterized as *C. parapsilosis* due to morphological similarities of the two species and has been reported to be a cause of invasive infections (43–45). The higher relative abundance of *C. orthopsilosis* in caesarean section-born infants shown in our results may have been due to their exposure to maternal antibiotics (Table 1). Because the growth of fungi is likely influenced by interkingdom interactions with bacterial community members (6), it is possible that birth mode-specific and differentially abundant bacteria may impact fungal community characteristics. For example, specific bacterial taxa, including *Streptococcaceae* and *Lactobacillales*, have been reported to have higher relative abundances in the oral cavity of infants with no maternal antibiotic exposure at the time of delivery (46), and species within these families have been shown to suppress biofilm formation by specific *Candida* species (47–50). Therefore, antibiotic administration may have allowed expansion of *C. orthopsilosis* in the oral cavity, or, because *Candida* species are often found in the hospital environment, antibiotic administration may have permitted higher rates of *C. orthopsilosis* acquisition from the environment (51–54). Due to the small number of subjects in this study, an analysis of bacterial (31) and fungal microbiome interactions was not completed and would likely have been underpowered with respect to detection of significant correlations. Longitudinal studies with large infant cohorts are needed in the future to understand the role of interkingdom relationships in microbiome and mycobiome development during infancy.

### Fungal variability within young infants.

In this study of early-life mycobiomes, we observed that the fungal communities of the skin, oral, and anal cavities were variable and that there was no clear progression toward a different or mature infant mycobiome composition (Fig. S2) during the first month of life. Additionally, for anal mycobiomes, early-life fungal compositions did not differ from those of adult females (Fig. S3). The anal and oral mycobiomes were diverse across infants and time, where within-infant beta diversity was not significantly lower than between-infant beta diversity (Fig. 3). In addition, mycobiome alpha diversity did not increase over time, regardless of body site, demonstrating a lack of new species acquisition and limited richness increases within the first month of life (Fig. 4). Unfortunately, there have been few longitudinal studies of very early (first month of life) bacterial microbiomes in humans that allow a comparison with our mycobiome results. One study, focused on bacterial communities of feces, observed that alpha diversity increases over the first month of life (55), in contrast to our findings determined with fungal communities. Longer-term studies of fecal microbiome development have shown that bacterial communities mature toward an adult-like state during the first 2 to 3 years of life (36, 56). Expanded longitudinal studies to similarly characterize mycobiome development are warranted. In particular, dense sampling in the earliest months of life, along with fungal and bacterial sequencing, would allow detection of key fungus-bacterium interactions that might prevent or enable colonization with specific taxa.

In summary, this report presents characteristics of mycobiome development during the first month of life. Initial body site differences, including the anal mycobiome having significantly more *C. albicans* and *C. parapsilosis* fungi than the skin and oral mycobiomes (Fig. 1a to c) and the oral mycobiome being significantly less diverse than the skin and anal mycobiomes (Fig. 2a), were detected. The skin community composition (beta diversity) was more stable than that of the oral and anal mycobiomes over time, where the skin mycobiome of each of the infants was more similar to that of the same infant over time than to those of other infants (Fig. 3a and b). Alpha diversity did not significantly increase over time (Fig. 4a), and we did not observe community composition maturation of the mycobiome over the first 30 days of life (Fig. S2a). Birth mode impacted the relative abundances of body site-specific taxa, including an increase in levels of skin-associated *C. albicans* in vaginally born infants and an increase in levels of orally associated *C. orthopsilosis* in infants born by caesarean section (Fig. 5c and e). These findings highlight the need for additional studies to better characterize the sources of and variability in fungal colonization early in life.

## MATERIALS AND METHODS

### Participant data.

Mothers (*n* = 17) who were pronounced to be healthy by their physicians were recruited from the University Hospital Puerto Rico Medical Center under approval from the Institutional Review Boards of the University of Puerto Rico Medical Sciences campus (approval A9710112) and from the Rio Piedras campus (approval 1011-107). Informed consent was obtained from all participants. All participants received standard-of-care treatment, such as prophylaxis antibiotics for mothers delivering by caesarean section and/or by vaginal birth if positive for group B *Streptococcus* (GBS) (Table 1).

### Sample collection and sequencing.

Samples from infants (*n* = 17) were collected with sterile swabs immediately following birth and again at days 3, 7, 14, 21, 27 and 30. Infant sample sites included the oral mucosa, forehead, and anal cavity. Samples from vaginal and anal sites of mothers (*n* = 16) were also collected immediately following the birth of the infant (Table 1). Of note, both infant and maternal anal samples were composed of both skin and fecal matter. Samples were placed on ice and frozen at −80°C within 2 h of collection until further processing was performed. DNA was extracted from thawed samples using a Mo Bio Powersoil kit (Mo Bio Laboratories, Inc., Carlsbad, CA). The ITS2 region of the fungal genome was amplified by PCR as previously described (5) with the following modifications. ITS2-specific oligonucleotide primers were modified to include a barcode of 6 bp for both the forward and the reverse primers to improve the amount of multiplexing performed on a single sequencing run. In addition, degenerate base pairs were included on the 5′ end of each primer to improve the quality of sequencing through increased length diversity and reductions in base pair homogeneity during photo acquisition. Finally, Kapa HiFi polymerase (Kapa Biosystems, Woburn, MA) was used to improve sequencing quality and reduce rates of PCR-related errors (57). ITS2 amplicons were generated under the following conditions: 95°C for 5 min; 30 cycles of 98°C for 20 s, 65°C for 15 s, and 72°C for 15 s; and a final extension of 72°C for 5 min. Amplicons were purified using a QIAQuick PCR purification kit (Qiagen, Germantown, MD) and eluted into 30 μl of PCR-grade water. Purified amplicons were quantitated with a Qubit 2.0 fluorometer (Invitrogen, Eugene, OR) using a Qubit double-stranded DNA (dsDNA) high-sensitivity (HS) assay kit (Invitrogen) and pooled for Illumina library construction using a TruSeq Nano kit (Illumina, San Diego, CA). Sequencing was performed at the University of Minnesota Genomics Center on an Illumina MiSeq system using a 2 × 250-bp paired-end version 2 MiSeq reagent kit (Illumina) as previously described.

### Mycobiome characterization.

Raw sequencing reads were preprocessed with SHI7 using the TruSeq3-2 adaptor trimming option (58). Primers were removed by trimming the first 25 bases of each read and truncating the sequences to a maximum read length of 150 bases. Reads shorter than 150 bases were dropped (see Table S3 in the supplemental material). Using a validated protocol, NINJA-OPS was used to align preprocessed reads against the UNITE v7 singleton-exclusive dynamic fungal ITS database release (31 January 2016) for NINJA-OPS using default options (59, 60). The resulting operational taxonomic unit (OTU) table was filtered to keep only those samples with at least 50 aligned reads and OTUs occurring in two or more samples (Table S3). OTU counts were converted to relative abundance data for all downstream analyses. Taxa correlating in relative abundance at a level above 95% across samples were collapsed into one taxon, decreasing the number of taxa from 123 to 96 across all samples. Alpha and beta diversity were calculated using QIIME v.1.8.0 (61), with the use of the dynamic 100 ghost-tree (0116_s) for phylogenetic metrics (62). Only one sample per individual, per time point, and per body site was retained for subsequent analysis, as some participants had multiple samples collected per day. For oral samples, only those taken prior to feeding were used for analysis, except in cases where only a sampling collected following feeding was available. Samples from feces and those listed as inoculated were dropped from the analysis due to inadequate sample size. Subsequent analysis of the data was performed in R, with the code available for download at the following URL: https://github.com/TonyaWard/fungal_infant1.

10.1128/mSystems.00140-17.8 TABLE S3  Sequencing processing outcomes. Download TABLE S3, DOCX file, 0.1 MB. Copyright © 2018 Ward et al. 2018 Ward et al. This content is distributed under the terms of the Creative Commons Attribution 4.0 International license.

To ensure that the levels of OTUs reported here were valid, a secondary analysis was performed using *de novo* OTU picking with the default setting of UCLUST (63) through QIIME v.1.8.0. The numbers of OTUs found with this method are reported in Table S2. Of note, a mean of 94% of *de novo* OTUs per sample failed to align to a known reference sequence.

### Statistical analyses.

Statistical analyses were performed in R. PERMANOVA was applied to test for differences in beta diversity centroids across sample types (Fig. 1a, 3, and 5a; see also Fig. S3a and S4b and c), and Wilcoxon rank sum tests were used to test for differences in the mean beta diversity distance from one sample type to another, including intra- and interinfant distances (Fig. 3 and 5b and d; see also Fig. S2). Testing for changes in alpha diversity (Fig. 4) and distance to self over time (Fig. S2) was performed using a permutation test of within-subject Spearman correlations. Student’s *t* tests were used to test for differences in alpha diversity across birth modes (within a time point and body site; Fig. S1) and across body sites (Fig. 2a). Testing for differentiated taxa across sample types was performed using a Kruskal-Wallis test for comparisons of data from more than two groups (across infant body sites, for example) or Wilcoxon rank sum tests for comparisons of data from two groups (delivery mode; Fig. 5c and e). Body site comparisons between specific sites were preformed pairwise with Wilcoxon rank sum tests with false-discovery-rate correction (Fig. 1d and e). Analyses comparing infant samples to maternal samples were not paired unless noted otherwise in the figure legend (Fig. 5; see also Fig. S4 and S5).

### Accession number(s).

Sequencing data are available under BioProject accession number PRJNA393442 within NCBI.

## References

[1] Mueller NT, Bakacs E, Combellick J, Grigoryan Z, Dominguez-Bello MG 2015 The infant microbiome development: mom matters. Trends Mol Med 21:109–117. doi:10.1016/j.molmed.2014.12.002. 25578246 PMC4464665

[2] Arrieta MC, Stiemsma LT, Dimitriu PA, Thorson L, Russell S, Yurist-Doutsch S, Kuzeljevic B, Gold MJ, Britton HM, Lefebvre DL, Subbarao P, Mandhane P, Becker A, McNagny KM, Sears MR, Kollmann T; CHILD Study, Mohn WW, Turvey SE, Finlay BB 2015 Early infancy microbial and metabolic alterations affect risk of childhood asthma. Sci Transl Med 7:307ra152. doi:10.1126/scitranslmed.aab2271. 26424567

[3] LaTuga MS, Ellis JC, Cotton CM, Goldberg RN, Wynn JL, Jackson RB, Seed PC 2011 Beyond bacteria: a study of the enteric microbial consortium in extremely low birth weight infants. PLoS One 6:e27858. doi:10.1371/journal.pone.0027858. 22174751 PMC3234235

[4] Strati F, Di Paola M, Stefanini I, Albanese D, Rizzetto L, Lionetti P, Calabrò A, Jousson O, Donati C, Cavalieri D, De Filippo C 2016 Age and gender affect the composition of fungal population of the human gastrointestinal tract. Front Microbiol 7:1227. doi:10.3389/fmicb.2016.01227. 27536299 PMC4971113

[5] Heisel T, Podgorski H, Staley CM, Knights D, Sadowsky MJ, Gale CA 2015 Complementary amplicon-based genomic approaches for the study of fungal communities in humans. PLoS One 10:e0116705. doi:10.1371/journal.pone.0116705. 25706290 PMC4338280

[6] Fujimura KE, Sitarik AR, Havstad S, Lin DL, Levan S, Fadrosh D, Panzer AR, LaMere B, Rackaityte E, Lukacs NW, Wegienka G, Boushey HA, Ownby DR, Zoratti EM, Levin AM, Johnson CC, Lynch SV 2016 Neonatal gut microbiota associates with childhood multisensitized atopy and T cell differentiation. Nat Med 22:1187–1191. doi:10.1038/nm.4176. 27618652 PMC5053876

[7] Wampach L, Heintz-Buschart A, Hogan A, Muller EEL, Narayanasamy S, Laczny CC, Hugerth LW, Bindl L, Bottu J, Andersson AF, de Beaufort C, Wilmes P 2017 Colonization and succession within the human gut microbiome by archaea, bacteria, and microeukaryotes during the first year of life. Front Microbiol 8:738. doi:10.3389/fmicb.2017.00738. 28512451 PMC5411419

[8] Bliss JM, Basavegowda KP, Watson WJ, Sheikh AU, Ryan RM 2008 Vertical and horizontal transmission of *Candida albicans* in very low birth weight infants using DNA fingerprinting techniques. Pediatr Infect Dis J 27:231–235. doi:10.1097/INF.0b013e31815bb69d. 18277930

[9] Nagata R, Nagano H, Ogishima D, Nakamura Y, Hiruma M, Sugita T 2012 Transmission of the major skin microbiota, *Malassezia*, from mother to neonate. Pediatr Int Off J Jpn Pediatr Soc 54:350–355. doi:10.1111/j.1442-200X.2012.03563.x. 22300401

[10] Kurtzman CP, Robnett CJ, Basehoar-Powers E 2008 Phylogenetic relationships among species of *Pichia*, *Issatchenkia* and *Williopsis* determined from multigene sequence analysis, and the proposal of *Barnettozyma* gen. nov., *Lindnera* gen. nov. and *Wickerhamomyces* gen. nov. FEMS Yeast Res 8:939–954. doi:10.1111/j.1567-1364.2008.00419.x. 18671746

[11] Borman AM, Linton CJ, Oliver D, Palmer MD, Szekely A, Johnson EM 2010 Rapid molecular identification of pathogenic yeasts by pyrosequencing analysis of 35 nucleotides of internal transcribed spacer 2. J Clin Microbiol 48:3648–3653. doi:10.1128/JCM.01071-10. 20702674 PMC2953101

[12] Wheeler ML, Limon JJ, Underhill DM 2017 Immunity to commensal fungi: detente and disease. Annu Rev Pathol 12:359–385. doi:10.1146/annurev-pathol-052016-100342. 28068483 PMC6573037

[13] Drell T, Lillsaar T, Tummeleht L, Simm J, Aaspõllu A, Väin E, Saarma I, Salumets A, Donders GGG, Metsis M 2013 Characterization of the vaginal micro- and mycobiome in asymptomatic reproductive-age Estonian women. PLoS One 8:e54379. doi:10.1371/journal.pone.0054379. 23372716 PMC3553157

[14] Schei K, Avershina E, Øien T, Rudi K, Follestad T, Salamati S, Ødegård RA 2017 Early gut mycobiota and mother-offspring transfer. Microbiome 5:107. doi:10.1186/s40168-017-0319-x. 28837002 PMC5571498

[15] Findley K, Oh J, Yang J, Conlan S, Deming C, Meyer JA, Schoenfeld D, Nomicos E, Park M; NIH Intramural Sequencing Center Comparative Sequencing Program, Kong HH, Segre JA 2013 Topographic diversity of fungal and bacterial communities in human skin. Nature 498:367–370. doi:10.1038/nature12171. 23698366 PMC3711185

[16] Zhang E, Tanaka T, Tajima M, Tsuboi R, Nishikawa A, Sugita T 2011 Characterization of the skin fungal microbiota in patients with atopic dermatitis and in healthy subjects. Microbiol Immunol 55:625–632. doi:10.1111/j.1348-0421.2011.00364.x. 21699559

[17] Jo JH, Deming C, Kennedy EA, Conlan S, Polley EC, Ng WI; NISC Comparative Sequencing Program, Segre JA, Kong HH 2016 Diverse human skin fungal communities in children converge in adulthood. J Invest Dermatol 136:2356–2363. doi:10.1016/j.jid.2016.05.130. 27476723 PMC5687974

[18] Jang SJ, Lim SH, Ko JH, Oh BH, Kim SM, Song YC, Yim SM, Lee YW, Choe YB, Ahn KJ 2009 The investigation on the distribution of *Malassezia* yeasts on the normal Korean skin by 26S rDNA PCR-RFLP. Ann Dermatol 21:18–26. doi:10.5021/ad.2009.21.1.18. 20548850 PMC2883363

[19] Sugita T, Suzuki M, Goto S, Nishikawa A, Hiruma M, Yamazaki T, Makimura K 2010 Quantitative analysis of the cutaneous *Malassezia* microbiota in 770 healthy Japanese by age and gender using a real-time PCR assay. Med Mycol 48:229–233. 19462267 10.1080/13693780902977976

[20] Faergemann J, Fredriksson T 1980 Age incidence of *Pityrosporum orbiculare* on human skin. Acta Derm Venereol 60:531–533. 6162342

[21] Gupta AK, Kohli Y 2004 Prevalence of *Malassezia* species on various body sites in clinically healthy subjects representing different age groups. Med Mycol 42:35–42. doi:10.1080/13693780310001610056. 14982112

[22] Ghannoum MA, Jurevic RJ, Mukherjee PK, Cui F, Sikaroodi M, Naqvi A, Gillevet PM 2010 Characterization of the oral fungal microbiome (mycobiome) in healthy individuals. PLoS Pathog 6:e1000713. doi:10.1371/journal.ppat.1000713. 20072605 PMC2795202

[23] Dupuy AK, David MS, Li L, Heider TN, Peterson JD, Montano EA, Dongari-Bagtzoglou A, Diaz PI, Strausbaugh LD 2014 Redefining the human oral mycobiome with improved practices in amplicon-based taxonomy: discovery of *Malassezia* as a prominent commensal. PLoS One 9:e90899. doi:10.1371/journal.pone.0090899. 24614173 PMC3948697

[24] Kleinegger CL, Lockhart SR, Vargas K, Soll DR 1996 Frequency, intensity, species, and strains of oral *Candida* vary as a function of host age. J Clin Microbiol 34:2246–2254. 8862593 10.1128/jcm.34.9.2246-2254.1996 PMC229226

[25] Russell C, Lay KM 1973 Natural history of *Candida* species and yeasts in the oral cavities of infants. Arch Oral Biol 18:957–962. doi:10.1016/0003-9969(73)90176-3. 4581575

[26] Chu DM, Ma J, Prince AL, Antony KM, Seferovic MD, Aagaard KM 2017 Maturation of the infant microbiome community structure and function across multiple body sites and in relation to mode of delivery. Nat Med 23:314–326. doi:10.1038/nm.4272. 28112736 PMC5345907

[27] Dominguez-Bello MG, Costello EK, Contreras M, Magris M, Hidalgo G, Fierer N, Knight R 2010 Delivery mode shapes the acquisition and structure of the initial microbiota across multiple body habitats in newborns. Proc Natl Acad Sci U S A 107:11971–11975. doi:10.1073/pnas.1002601107. 20566857 PMC2900693

[28] Costello EK, Carlisle EM, Bik EM, Morowitz MJ, Relman DA 2013 Microbiome assembly across multiple body sites in low-birthweight infants. mBio 4:e00782-13. doi:10.1128/mBio.00782-13. 24169577 PMC3809564

[29] Chin VK, Lee TY, Rusliza B, Chong PP 2016 Dissecting *Candida albicans* infection from the perspective of *C. albicans* virulence and omics approaches on host-pathogen interaction: a review. Int J Mol Sci 17:1643. doi:10.3390/ijms17101643. PMC5085676 27763544

[30] Mayer FL, Wilson D, Hube B 2013 *Candida albicans* pathogenicity mechanisms. Virulence 4:119–128. doi:10.4161/viru.22913. 23302789 PMC3654610

[31] Dominguez-Bello MG, De Jesus-Laboy KM, Shen N, Cox LM, Amir A, Gonzalez A, Bokulich NA, Song SJ, Hoashi M, Rivera-Vinas JI, Mendez K, Knight R, Clemente JC 2016 Partial restoration of the microbiota of cesarean-born infants via vaginal microbial transfer. Nat Med 22:250–253. doi:10.1038/nm.4039. 26828196 PMC5062956

[32] Barbosa JO, Rossoni RD, Vilela SFG, de Alvarenga JA, Velloso Mdos S, Prata MC, Jorge AO, Junqueira JC 2016 Streptococcus mutans can modulate biofilm formation and attenuate the virulence of *Candida albicans*. PLoS One 11:e0150457. doi:10.1371/journal.pone.0150457. 26934196 PMC4774980

[33] Parolin C, Marangoni A, Laghi L, Foschi C, Ñahui Palomino RA, Calonghi N, Cevenini R, Vitali B 2015 Isolation of vaginal *Lactobacilli* and characterization of anti-*Candida* activity. PLoS One 10:e0131220. doi:10.1371/journal.pone.0131220. 26098675 PMC4476673

[34] Sharma A, Srivastava S 2014 Anti-Candida activity of spent culture filtrate of *Lactobacillus plantarum* strain LR/14. J Mycol Med 24:e25–e34. doi:10.1016/j.mycmed.2013.11.001. 24316318

[35] Köhler GA, Assefa S, Reid G 2012 Probiotic interference of *Lactobacillus rhamnosus* GR-1 and *Lactobacillus reuteri* RC-14 with the opportunistic fungal pathogen *Candida albicans*. Infect Dis Obstet Gynecol 2012:e636474. doi:10.1155/2012/636474. PMC3395238 22811591

[36] Bokulich NA, Chung J, Battaglia T, Henderson N, Jay M, Li H, D Lieber AD, Wu F, Perez-Perez GI, Chen Y, Schweizer W, Zheng X, Contreras M, Dominguez-Bello MG, Blaser MJ 2016 Antibiotics, birth mode, and diet shape microbiome maturation during early life. Sci Transl Med 8:343ra82. doi:10.1126/scitranslmed.aad7121. PMC5308924 27306664

[37] Yassour M, Vatanen T, Siljander H, Hämäläinen AM, Härkönen T, Ryhänen SJ, Franzosa EA, Vlamakis H, Huttenhower C, Gevers D, Lander ES, Knip M; DIABIMMUNE Study Group, Xavier RJ 2016 Natural history of the infant gut microbiome and impact of antibiotic treatment on bacterial strain diversity and stability. Sci Transl Med 8:343ra81. doi:10.1126/scitranslmed.aad0917. PMC5032909 27306663

[38] Payne MS, Cullinane M, Garland SM, Tabrizi SN, Donath SM, Bennett CM, Amir LH 2016 Detection of *Candida* spp. in the vagina of a cohort of nulliparous pregnant women by culture and molecular methods: is there an association between maternal vaginal and infant oral colonisation? Aust N Z J Obstet Gynaecol 56:179–184. doi:10.1111/ajo.12409. 26437337

[39] Leung MHY, Chan KCK, Lee PKH 2016 Skin fungal community and its correlation with bacterial community of urban Chinese individuals. Microbiome 4:46. doi:10.1186/s40168-016-0192-z. 27558504 PMC4997687

[40] Park HK, Ha MH, Park SG, Kim MN, Kim BJ, Kim W 2012 Characterization of the fungal microbiota (mycobiome) in healthy and dandruff-afflicted human scalps. PLoS One 7:e32847. doi:10.1371/journal.pone.0032847. 22393454 PMC3290624

[41] Bellemain E, Carlsen T, Brochmann C, Coissac E, Taberlet P, Kauserud H 2010 ITS as an environmental DNA barcode for fungi: an in silico approach reveals potential PCR biases. BMC Microbiol 10:189. doi:10.1186/1471-2180-10-189. 20618939 PMC2909996

[42] Bazzicalupo AL, Bálint M, Schmitt I 2013 Comparison of ITS1 and ITS2 rDNA in 454 sequencing of hyperdiverse fungal communities. Fungal Ecol 6:102–109. doi:10.1016/j.funeco.2012.09.003.

[43] Tavanti A, Davidson AD, Gow NAR, Maiden MCJ, Odds FC 2005 *Candida orthopsilosis* and *Candida metapsilosis* spp. nov. to replace *Candida parapsilosis* groups II and III. J Clin Microbiol 43:284–292. doi:10.1128/JCM.43.1.284-292.2005. 15634984 PMC540126

[44] Bonfietti LX, Martins Mdos A, Szeszs MW, Pukiskas SB, Purisco SU, Pimentel FC, Pereira GH, Silva DC, Oliveira L, Carvalho Melhem MDS 2012 Prevalence, distribution and antifungal susceptibility profiles of *Candida parapsilosis*, *Candida orthopsilosis* and *Candida metapsilosis* bloodstream isolates. J Med Microbiol 61:1003–1008. doi:10.1099/jmm.0.037812-0. 22493277

[45] Lovero G, Borghi E, Balbino S, Cirasola D, De Giglio O, Perdoni F, Caggiano G, Morace G, Montagna MT 2016 Molecular identification and echinocandin susceptibility of *Candida parapsilosis* complex bloodstream isolates in Italy, 2007–2014. PLoS One 11:e0150218. doi:10.1371/journal.pone.0150218. 26919294 PMC4769087

[46] Gomez-Arango LF, Barrett HL, McIntyre HD, Callaway LK, Morrison M, Dekker Nitert M 2017 Antibiotic treatment at delivery shapes the initial oral microbiome in neonates. Sci Rep 7:43481. doi:10.1038/srep43481. 28240736 PMC5378909

[47] Chandra J, Mukherjee PK 2015 *Candida* biofilms: development, architecture, and resistance. Microbiol Spectr 3:10. doi:10.1128/microbiolspec.MB-0020-2015. PMC4566167 26350306

[48] Reid G, Tieszer C, Lam D 1995 Influence of *Lactobacilli* on the adhesion of *Staphylococcus aureus* and *Candida albicans* to fibers and epithelial cells. J Ind Microbiol 15:248–253. doi:10.1007/BF01569832. 8519484

[49] Webb BC, Willcox MD, Thomas CJ, Harty DW, Knox KW 1995 The effect of sodium hypochlorite on potential pathogenic traits of *Candida albicans* and other *Candida* species. Oral Microbiol Immunol 10:334–341. doi:10.1111/j.1399-302X.1995.tb00163.x. 8602340

[50] Holmes AR, Cannon RD, Jenkinson HF 1995 Interactions of *Candida albicans* with bacteria and salivary molecules in oral biofilms. J Ind Microbiol 15:208–213. doi:10.1007/BF01569827. 8519479

[51] Sabino R, Sampaio P, Carneiro C, Rosado L, Pais C 2011 Isolates from hospital environments are the most virulent of the Candida parapsilosis complex. BMC Microbiol 11:180. doi:10.1186/1471-2180-11-180. 21824396 PMC3166928

[52] Sautour M, Dalle F, Olivieri C, L’ollivier C, Enderlin E, Salome E, Chovelon I, Vagner O, Sixt N, Fricker-Pap V, Aho S, Fontaneau O, Cachia C, Bonnin A 2009 A prospective survey of air and surface fungal contamination in a medical mycology laboratory at a tertiary care university hospital. Am J Infect Control 37:189–194. doi:10.1016/j.ajic.2008.06.009. 19059674

[53] van Asbeck EC, Huang YC, Markham AN, Clemons KV, Stevens DA 2007 *Candida parapsilosis* fungemia in neonates: genotyping results suggest healthcare workers hands as source, and review of published studies. Mycopathologia 164:287–293. doi:10.1007/s11046-007-9054-3. 17874281

[54] Welsh RM, Bentz ML, Shams A, Houston H, Lyons A, Rose LJ, Litvintseva AP 2017 Survival, persistence, and isolation of the emerging multidrug-resistant pathogenic yeast *Candida auris* on a plastic health care surface. J Clin Microbiol 55:2996–3005. doi:10.1128/JCM.00921-17. 28747370 PMC5625385

[55] Hill CJ, Lynch DB, Murphy K, Ulaszewska M, Jeffery IB, O’Shea CA, Watkins C, Dempsey E, Mattivi F, Tuohy K, Ross RP, Ryan CA, O’Toole PW, Stanton C 2017 Evolution of gut microbiota composition from birth to 24 weeks in the INFANTMET Cohort. Microbiome 5:21. doi:10.1186/s40168-017-0240-3. 28095889 PMC5240274

[56] Yatsunenko T, Rey FE, Manary MJ, Trehan I, Dominguez-Bello MG, Contreras M, Magris M, Hidalgo G, Baldassano RN, Anokhin AP, Heath AC, Warner B, Reeder J, Kuczynski J, Caporaso JG, Lozupone CA, Lauber C, Clemente JC, Knights D, Knight R, Gordon JI 2012 Human gut microbiome viewed across age and geography. Nature 486:222–227. doi:10.1038/nature11053. 22699611 PMC3376388

[57] Gohl DM, Vangay P, Garbe J, MacLean A, Hauge A, Becker A, Gould TJ, Clayton JB, Johnson TJ, Hunter R, Knights D, Beckman KB 2016 Systematic improvement of amplicon marker gene methods for increased accuracy in microbiome studies. Nat Biotechnol 34:942–949. doi:10.1038/nbt.3601. 27454739

[58] Al-Ghalith GA, Ang K, Hillmann B, Knights D 2017 SHI7: a streamlined short-read iterative trimming pipeline. https://zenodo.org. doi:10.5281/zenodo.808832.

[59] Al-Ghalith GA, Montassier E, Ward HN, Knights D 2016 NINJA-OPS: fast accurate marker gene alignment using concatenated ribosomes. PLoS Comput Biol 12:e1004658. doi:10.1371/journal.pcbi.1004658. 26820746 PMC4731464

[60] Cline LC, Song Z, Al-Ghalith GA, Knights D, Kennedy PG 2017 Moving beyond de novo clustering in fungal community ecology. New Phytol 216:629–634. doi:10.1111/nph.14752. 28782807

[61] Caporaso JG, Kuczynski J, Stombaugh J, Bittinger K, Bushman FD, Costello EK, Fierer N, Peña AG, Goodrich JK, Gordon JI, Huttley GA, Kelley ST, Knights D, Koenig JE, Ley RE, Lozupone CA, McDonald D, Muegge BD, Pirrung M, Reeder J, Sevinsky JR, Turnbaugh PJ, Walters WA, Widmann J, Yatsunenko T, Zaneveld J, Knight R 2010 QIIME allows analysis of high-throughput community sequencing data. Nat Methods 7:335–336. doi:10.1038/nmeth.f.303. 20383131 PMC3156573

[62] Fouquier J, Rideout JR, Bolyen E, Chase J, Shiffer A, McDonald D, Knight R, Caporaso JG, Kelley ST 2016 Ghost-tree: creating hybrid-gene phylogenetic trees for diversity analyses. Microbiome 4:11. doi:10.1186/s40168-016-0153-6. 26905735 PMC4765138

[63] Edgar RC 2010 Search and clustering orders of magnitude faster than BLAST. Bioinformatics 26:2460–2461. doi:10.1093/bioinformatics/btq461. 20709691

