# Supplementary figures and images for: Development of the Human Mycobiome over the First Month of Life and across Body Sites
Source: mSystems. 2018 Mar 6;3(3):e00140-17. doi: 10.1128/mSystems.00140-17 (PMC5840654; doi:10.1128/mSystems.00140-17)

**a**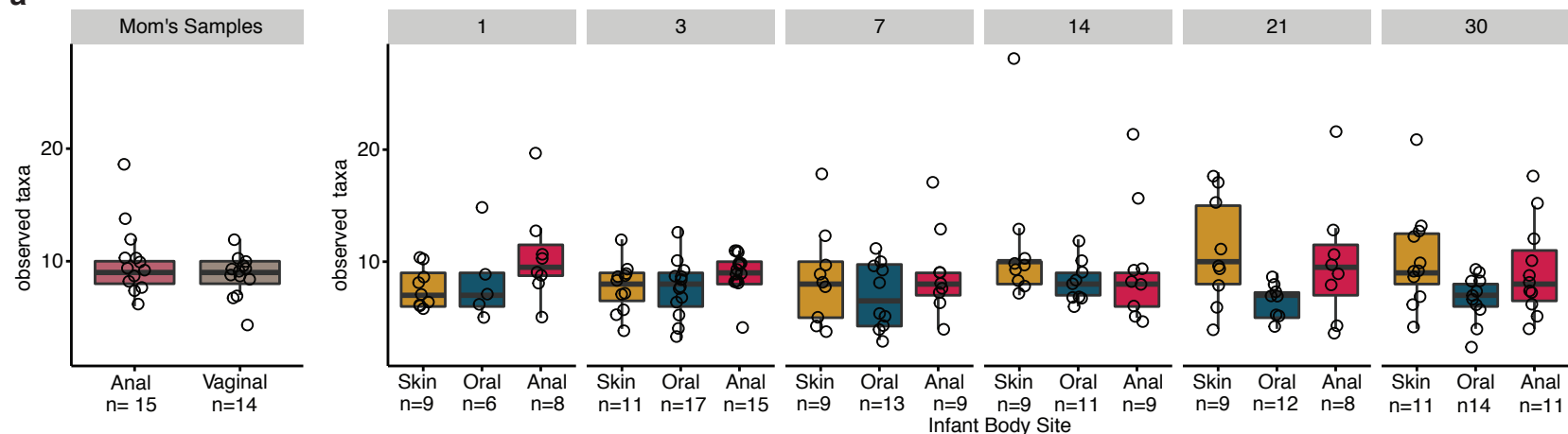**b**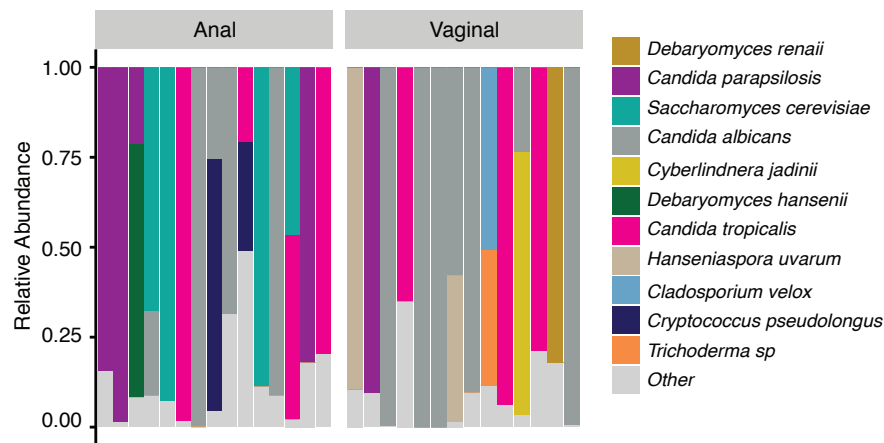**c**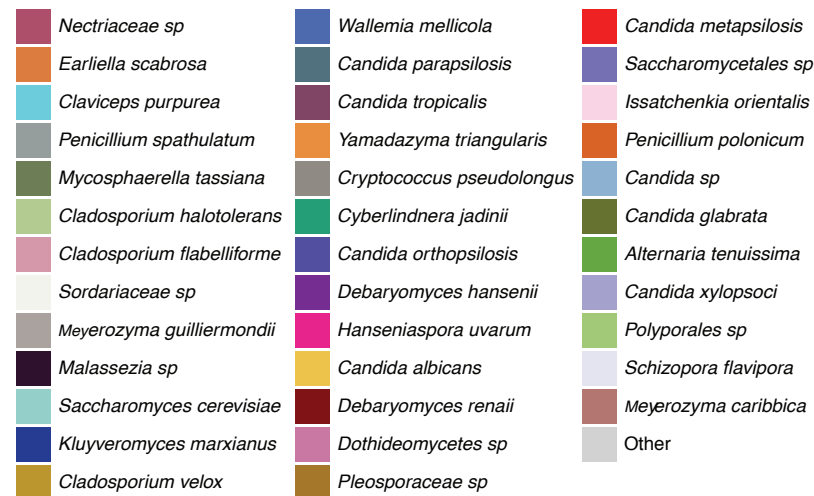

Supplement: FIG S1 [file sys001182203sf1.pdf]

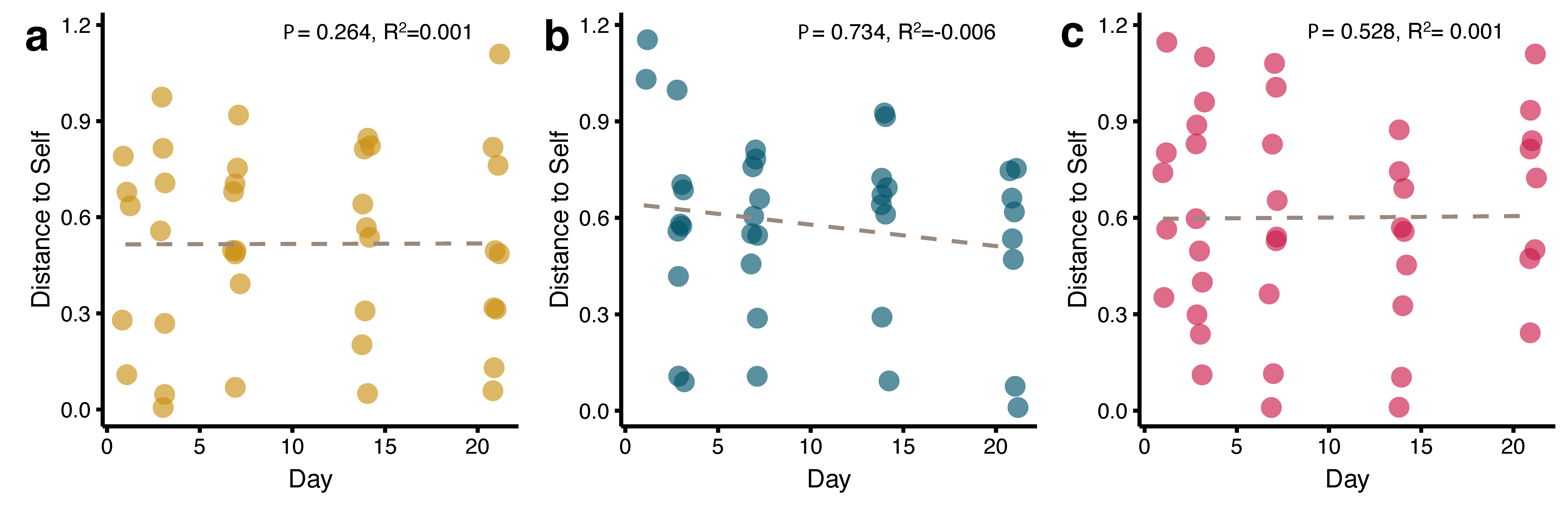

Supplement: FIG S2 [file sys001182203sf2.tif]

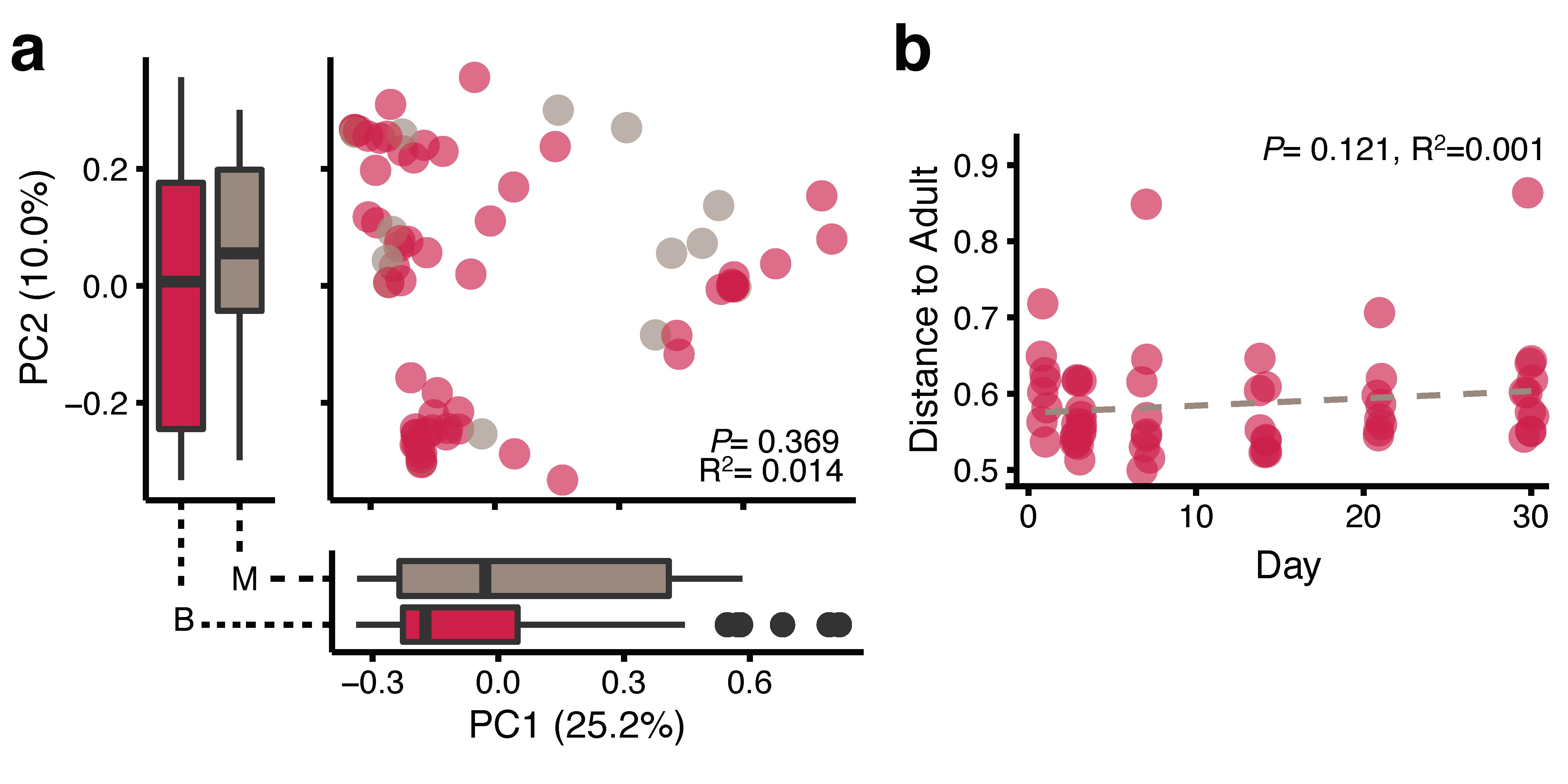

Supplement: FIG S3 [file sys001182203sf3.tif]

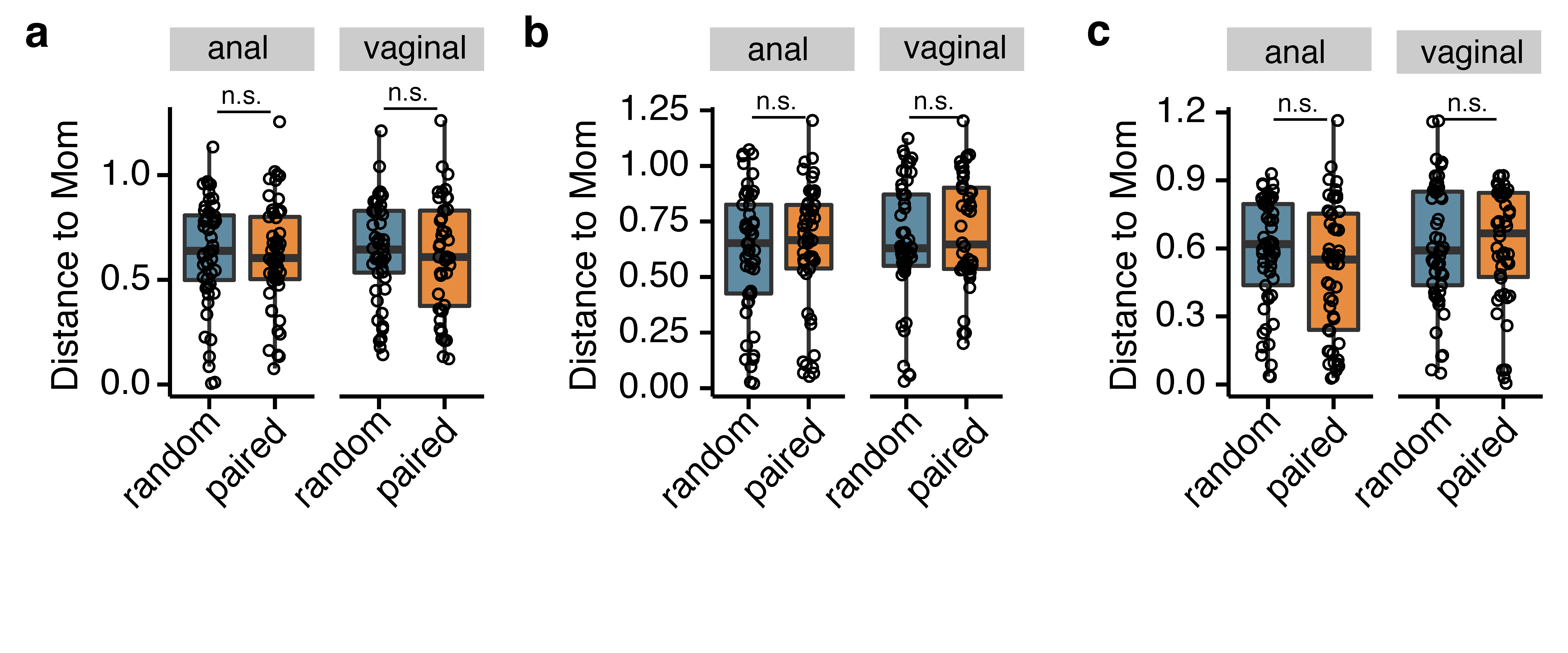

Supplement: FIG S4 [file sys001182203sf4.tif]

**a**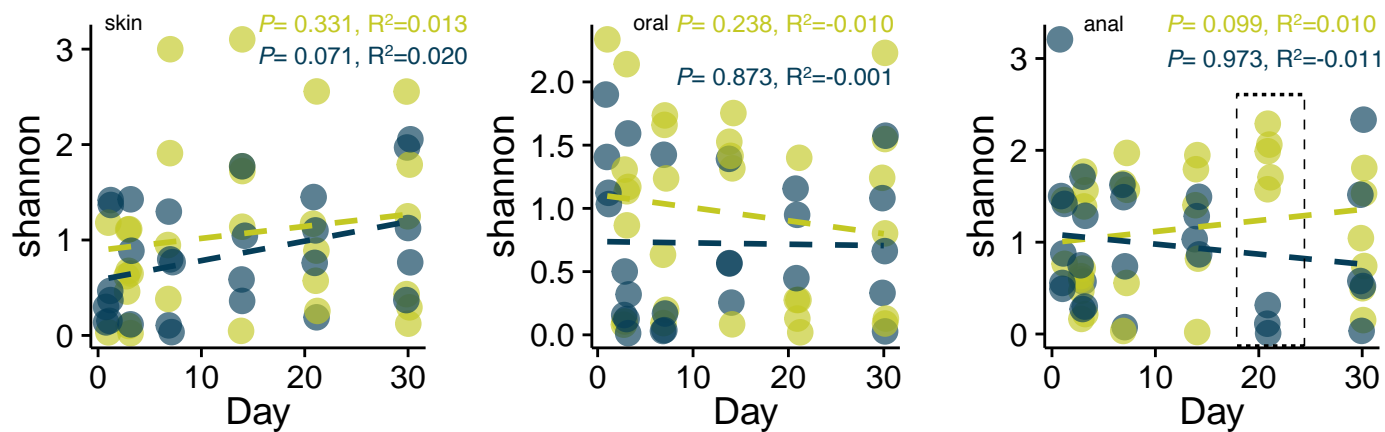**b**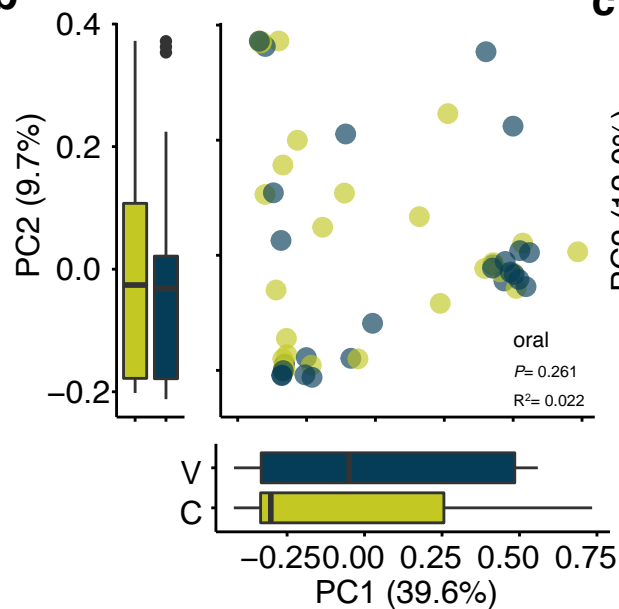**c**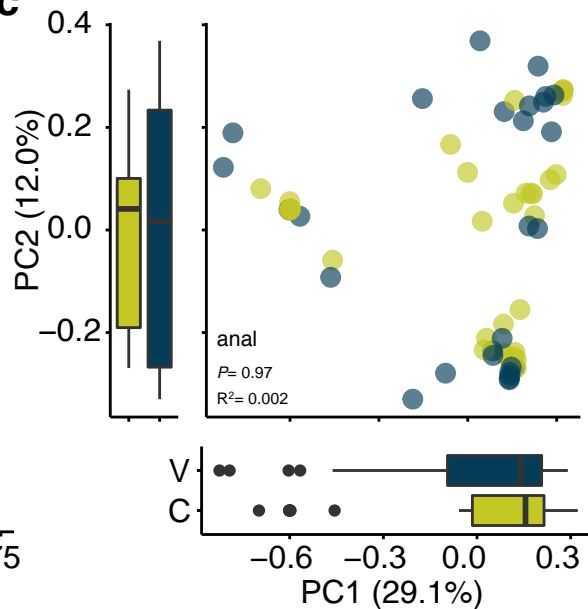**d**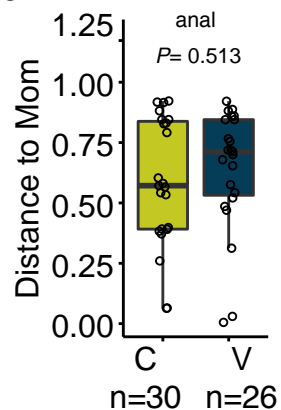

Supplement: FIG S5 [file sys001182203sf5.pdf]
