# Supplementary material for: Development of the Human Mycobiome over the First Month of Life and across Body Sites
Source: mSystems. 2018 Mar 6;3(3):e00140-17. doi: 10.1128/mSystems.00140-17 (PMC5840654; doi:10.1128/mSystems.00140-17)
Supplement: TABLE S1 [file sys001182203st1.docx]

**Supplemental Table 1. Top five most abundant infant and maternal fungal taxa by body site.**

| Sample Origin | Body Site | Taxon | Mean Relative Abundance |
| --- | --- | --- | --- |
| Infant |  |  |  |
|  | **Skin** | *Candida tropicalis* | 20.84 |
|  |  | *C. parapsilosis* | 16.4 |
|  |  | *Saccharomyces cerevisiae* | 14.83 |
|  |  | *C. albicans* | 6.14 |
|  |  | *C. orthopsilosis* | 5.18 |
|  | **Oral** | *C. parapsilosis* | 25.52 |
|  |  | *C. tropicalis* | 14.54 |
|  |  | *S. cerevisiae* | 11.04 |
|  |  | *C. orthopsilosis* | 10.05 |
|  |  | *Cladosporium velox* | 7.58 |
|  | **Anal** | *C. parapsilosis* | 32.24 |
|  |  | *C. tropicalis* | 23.8 |
|  |  | *C. albicans* | 15.13 |
|  |  | *S. cerevisiae* | 11.97 |
|  |  | *Cryptococcus pseudolongus* | 4.55 |
| Maternal |  |  |  |
|  | **Vaginal** | *C. albicans* | 44.87 |
|  |  | *C. tropicalis* | 17.75 |
|  |  | *Hanseniaspora uvarum* | 9.3 |
|  |  | *C. parapsilosis* | 8.19 |
|  |  | *Debaryomyces renaii* | 5.61 |
|  | **Anal** | *C. albicans* | 23.15 |
|  |  | *S. cerevisiae* | 21.41 |
|  |  | *C. parapsilosis* | 20.29 |
|  |  | *C. tropicalis* | 19.23 |
|  |  | *C. pseudolongus* | 6.77 |
