# Supplementary material for: Development of the Human Mycobiome over the First Month of Life and across Body Sites
Source: mSystems. 2018 Mar 6;3(3):e00140-17. doi: 10.1128/mSystems.00140-17 (PMC5840654; doi:10.1128/mSystems.00140-17)
Supplement: TABLE S2 [file sys001182203st2.docx]

**Supplemental Table 2. Top five most prevalent infant and maternal fungal taxa by**

**body site.**

| Sample Origin | Body Site | Taxon | % Samples |
| --- | --- | --- | --- |
| Infant |  |  |  |
|  | **Skin** | *Candida parapsilosis* | 89.6 |
|  |  | *C. tropicalis* | 87.9 |
|  |  | *Saccharomyces cerevisiae* | 77.6 |
|  |  | *C. albicans* | 55.2 |
|  |  | *C. orthopsilosis* | 39.6 |
|  | **Oral** | *C. parapsilosis* | 96.4 |
|  |  | *C. tropicalis* | 89.3 |
|  |  | *S. cerevisiae* | 64.3 |
|  |  | *C. orthopsilosis* | 62.5 |
|  |  | *C. albicans* | 57.1 |
|  | **Anal** | *C. parapsilosis* | 95.0 |
|  |  | *C. tropicalis* | 93.3 |
|  |  | *C. albicans* | 85.0 |
|  |  | *S. cerevisiae* | 83.3 |
|  |  | *C. orthopsilosis* | 61.7 |
| Maternal |  |  |  |
|  | **Vaginal** | *C. albicans* | 100 |
|  |  | *C. parapsilosis* | 100 |
|  |  | *C. tropicalis* | 85.7 |
|  |  | *S. cerevisiae* | 85.7 |
|  |  | *Cladosporium velox* | 42.8 |
|  | **Anal** | *C. albicans* | 100 |
|  |  | *C. parapsilosis* | 100 |
|  |  | *C. tropicalis* | 100 |
|  |  | *S. cerevisiae* | 86.7 |
|  |  | *C. orthopsilosis* | 84.6 |
