# Supplementary material for: Development of the Human Mycobiome over the First Month of Life and across Body Sites
Source: mSystems. 2018 Mar 6;3(3):e00140-17. doi: 10.1128/mSystems.00140-17 (PMC5840654; doi:10.1128/mSystems.00140-17)
Supplement: TABLE S3 [file sys001182203st3.docx]

**Supplemental Table 3. Sequencing processing outcomes.**

| Body Site | No.  of Samples | Raw Reads  (mean +/- SD) | Reads Passing QC  (mean +/- SD) | Reads Aligning  (mean +/- SD) | Samples Dropped  (< 50 aligned) | OTUs de novo  (mean +/- SD) | OTUs Closed  (mean +/- SD) | Taxa  (mean +/- SD) |
| --- | --- | --- | --- | --- | --- | --- | --- | --- |
| Skin (infant) | 65 | 22,882 +/- 26,006 | 6,488 +/- 12,098 | 1,866 +/- 6,407 | 2 | 1,651 +/- 1,493 | 10 +/- 6 | 10 +/- 5 |
| Oral (infant) | 90 | 19,616 +/- 21,738 | 5,404 +/- 11,010 | 983 +/- 6,092 | 16 | 1,705 +/- 1,494 | 8 +/- 3 | 8 +/- 3 |
| Anal (infant) | 67 | 34,458 +/- 31,474 | 9,216 +/- 17,315 | 1,784 +/- 6,723 | 1 | 2,420 +/- 1,620 | 10 +/- 5 | 10 +/- 4 |
| Anal (maternal) | 16 | 30,464 +/- 24,147 | 7,010 +/- 11,776 | 604 +/- 788 | 1 | 2,206 +/- 1,445 | 10 +/- 4 | 10 +/- 4 |
| Vaginal (maternal) | 16 | 71,003 +/- 158,498 | 60,331 +/- 158,743 | 2,548 +/- 44,29 | 2 | 1,269 +/- 1,555 | 9 +/- 3 | 10 +/- 2 |
